# Prevalence of disrespect and abuse during facility based child birth and associated factors, Jimma University Medical Center, Southwest Ethiopia

**DOI:** 10.1186/s12884-019-2332-5

**Published:** 2019-05-27

**Authors:** Ahmed Siraj, Woubishet Teka, Habtemu Hebo

**Affiliations:** 1 Shenen Gibe Hospital, Jimma, Ethiopia; 2 Department of Gynecology & Obstetrics, Medical Science Faculty, Institute of Health, Jimma University, Jimma, Ethiopia; 3 Department of Epidemiology, Public Health Faculty, Institute of Health, Jimma University, Jimma, Ethiopia

**Keywords:** Disrespect, Abuse, Delivery, Childbirth, Facility-based, Ethiopia

## Abstract

**Background:**

In countries where the proportion of births attended by skilled providers is low, maternal mortality is high. According to the 2016 EDHS report, the proportion of births attended by skilled providers was only 26% and the maternal mortality ratio was 412 per 100,000 live-births. Disrespectful and abusive behavior of health workers and other facility staff experienced by women during facility-based childbirth is important, but the little-understood barrier of institutional delivery.

**Objective:**

This study assessed the prevalence of disrespect and abuse experienced by mothers during facility-based childbirth and associated factors.

**Methods:**

A facility based cross-sectional study was undertaken from October to December 2016. Data were collected by face-to-face interview using a structured questionnaire from 290 mothers consecutively included in the study immediately prior to discharge from the hospital. Reports of disrespect and abuse during childbirth were measured using 23 performance indicators. Data were entered into EpiData and analyzed by SPSS; bivariate and multivariable binary logistic regression analyses were performed to identify factors associated with disrespect and abuse.

**Result:**

Three-fourths **(**217,[74.8%]) of participants were Muslim. Nearly half (142,[49%]) had a primary level of education. Most (232,[80%]) were housewives and 175(60.3%) were from outside Jimma town. The prevalence of disrespect and abuse during childbirth was 91.7% (266/290; 95%CI:0.879,0.946). The most common types of disrespect and abuse reported were culturally inappropriate care (218,[75.2%]), failure to encourage the client to ask questions (220,[75.9%]), the provider not introducing him/herself (232,[80.0%]), failure to obtain consent/permission prior to any procedure (185,[63.8%]) and not using curtains/visual barriers to protect client (237,[81.7%]). Being non-married [95%CI:(0.009,0.222), ≥para-II [95%CI:(0.093,0.862)] and being attended by female care provider [95%CI:(0.026,0.224)] were associated with the reduced chance of reporting disrespect and abuse. However, achieving ≥secondary education [95%CI:(1.028,10.272)] was associated with a higher chance of reporting disrespect and abuse.

**Conclusion:**

The very high prevalence of abuse or disrespect during facility-based delivery shows a health system in crisis. A key implication of this finding is that efforts to increase facility-based delivery must address disrespect and abuse to ensure higher utilization by women. Making facility-based deliveries attended by female providers may reduce the problem.

## Plain English summary

In countries where the proportion of births attended by skilled providers is low, maternal mortality is high. According to the 2016 Ethiopian Demographic and Health Surveillance report, the proportion of births attended by skilled providers was only 26% and the maternal mortality ratio was 412 per 100,000 live-births. Disrespectful and abusive behavior of health workers and other facility staff experienced by women during facility-based childbirth is important, but the little-understood barrier of institutional delivery. This study assessed the prevalence of disrespect and abuse experienced by mothers during facility-based childbirth and associated factors.

October to December 2016, consecutively included 290 mothers were interviewed immediately prior to discharge from the hospital. The result showed that nearly 92 out of 100 mothers were disrespected and abused during childbirth. Three-fourths of mothers did not receive culturally appropriate care and were not encouraged to ask questions. The provider did not introduce him/herself to four-fifths of mothers. The provider also did not request consent or permission prior to any procedure from nearly two-thirds of mothers. The provider did not use curtains or other visual barriers to protect client for more than four-fifths of mothers. Non-married mother, para-II or above mother and mother attended by female care provider had reduced chance of reporting disrespect and abuse. However, mother who attended at least secondary school had a higher chance of reporting disrespect and abuse.

In conclusion, the magnitude of disrespect and abuse during childbirth was very high in the study area indicating a health system in crisis. A key implication of this finding is that efforts to increase facility delivery must address disrespect and abuse to ensure higher utilization by women. Making facility-based deliveries attended by female providers may reduce the problem.

## Background

In countries where the proportion of births attended by skilled providers is low, maternal mortality is high [1]. Reducing the global maternal mortality ratio to less than 70 per 100,000 live births by 2030 is the first target of Sustainable Development Goal (SDG) 3. The proportion of births attended by skilled health personnel is a critical progress indicator explicitly adopted for this target [2, 3]. Ethiopia had a plan to decrease the maternal mortality ratio (MMR) from 871 to 267 maternal deaths per 100,000 live births between 1990 and 2015 to achieve the millennium development goal (MDG) 5. A key indicator of this achievement was the proportion of births attended by skilled health personnel [4]. However, according to the 2016 Ethiopian Demographic and Health Surveillance (EDHS) report, maternal mortality ratio was 412 deaths per 100,000 live births [5]. Although the coverage of trained midwives increased to 72.7% in fiscal year 2015/16 [6], the proportion of births attended by skilled providers was low (26%) [5].

Lack of respect and courtesy from providers, perceived poor service quality, fear to expose the body to strangers, perceived costs of using health facility, and fear of being attended by male providers during birth are all known to contribute to low institutional delivery rates [7–15]. In Addis Ababa, the capital city of Ethiopia, though the proportion of births occurring in health institutions is much higher than the national Fig. (82.3%), there are growing concerns about the respect and friendliness of safe delivery services [16].

Disrespectful and abusive behavior by health workers and other facility staff is important, but the little-understood component of the poor quality care experienced by women during childbirth in facilities. Non-abusive and respectful care at birth encompasses many points along a continuum spanning patient-centered and dignified care to overtly abusive and non-dignified maternal care. Maternal health experts and many stakeholders agree that disrespect and abuse (D&A) during facility-based childbirth represent important causes of suffering for women and are important barriers to women choosing to access skilled care [17, 18]. However, disrespect and abuse are often multi-factorial and may be perceived differently (or even normalized) depending on the specific setting.

According to Bowser and Hill’s comprehensive review, the seven categories of disrespect and abuse during childbirth are physical abuse, non-dignified care, discrimination based on specific patient attributes, non-consented care, non-confidential care, abandonment of care and detention in facilities. However, it is known that manifestations of abuse and disrespect often fall into more than one category, and these categories are not intended to be mutually exclusive. Categories should be rather seen as overlapping and representing a continuum. Numerous factors (individual and community-level) may contribute to the experiences of disrespect and abuse. Lack of legal and ethical foundations to address D&A, normalizing D&A, lack of standards and accountability, lack of leadership commitment, and provider prejudice due to training and lack of resources are some among many factors [17, 18].

Disrespect and abuse of women during childbirth at health facilities have been qualitatively described, but there is little quantitative evidence on the prevalence of D&A during facility-based maternity services delivered in low-resource settings. Few recent studies conducted in Kenya, Tanzania, Ethiopia, and Nigeria analyzed women’s experiences during childbirth and estimated prevalence of disrespect and abuse that ranged from 20 to 98% [19–24]. Having a good understanding of the prevalence of D&A and factors that influence it is, however, critical for developing interventions and encouraging clients’ future facility utilization. This study, thus, aimed at quantitatively describing the level and types of disrespect and abuse women faced during facility-based childbirth.

## Methods

### Study area and period

The study was conducted in Jimma University Medical Centre (JUMC) from October to December 2016. JUMC is located in Jimma town, 352 Km Southwest of Addis Ababa. JUMC is one of teaching medical centers in the country giving services to people living in Jimma zone and serving as a referral in Southwest Ethiopia. It is also serving as a clinical postgraduate specialty teaching hospital for Obstetrics and Gynecology, Internal Medicine, Pediatrics & Child Health since 2005 and for Ophthalmology, Anesthesiology, and Surgery since 2007. Department of Obstetrics and Gynecology had 8 consultant Obstetricians & Gynecologists and 33 residents (year I – IV), some midwives and nurses providing services. There were also final year under-graduate medicine students (Medical Interns), midwifery and nursing students providing services under supervision.

### Study design

A quantitative cross-sectional study using interviewer-administered questionnaire was conducted to measure the prevalence of disrespect and abuse during facility-based childbirth and associated factors.

### Study population

All women who gave birth vaginally at JUMC during the study period and have been given consent were recruited for the study. Mothers who gave birth with cesarean section were excluded for two reasons; to rule out the effect of anesthesia, and to minimize the time lapse between childbirth and time of interview.

### Sample size determination and sampling technique

A single population proportion formula was used to estimate the sample size with assumptions of 78.6% prevalence of any D&A [21], 5% margin of error, 95% confidence level and 10% non-response rate.

$$ \mathrm{n}=\frac{\left(\mathrm{Z}\upalpha /2\right)\ast \left(\mathrm{P}\ast \mathrm{Q}\right)}{\mathrm{d}2}=\frac{{\left(1.96\ \right)}^2\ast \left(0.786\ast 0.214\right)}{(0.05)2}=258 $$

Adding 10% for non-response, the final sample size became 284. Study participants were selected by convenient consecutive sampling technique until the required sample size was achieved.

### Variables

Dependent variables: prevalence of disrespect and abuse (D&A).

Independent variables: socio-demographic/economic characteristics [age, religion, ethnicity, marital status, educational status, address (Jimma town, Out of Jimma town), monthly income] and obstetric and service-related factors [parity, ANC use, history of institutional delivery, health provider (sex, number), length of stay, complication during delivery].

### Data collection and measurement

Data were collected using structured questionnaire prepared after reviewing related literature. The questionnaire was translated into local languages (Amharic and Afan Oromo) and back-translated to English to ensure consistency of translation. A total of 39 questions (16 background and 23 D&A questions) comprised the questionnaire and most questions were closed-ended (yes/no or multiple choice) (Table S1). The average time required to complete the questionnaire was 30 min.

Data were collected by face-to-face interview (the data collectors asked the women the questions and they logged their responses) immediately prior to discharge from the health facility after childbirth. Data collection was delayed until the time of discharge from the hospital to reduce social desirability and recall biases. The soonest data collection was 6 h after childbirth. The interview was conducted in a quiet, private area of the unit with one woman and one interviewer. Four female nurses not involved in the women’s care were recruited for data collection and trained for one day on how to use the data collection tool before embarking on data collection. Disrespect and abuse was measured by 23 verification criteria under major seven categories. The tool was adapted from the Maternal and Child Health Integrated Program (MCHIP) who developed it as part of its respectful maternity care toolkit [25].

### Data processing and analysis

Data were entered into EpiInfo and analyzed using SPSS version 20 software. Descriptive statistics were computed. Adequacies of cells were checked in chi-square test for each independent variable. Bivariate binary logistic regression was performed for variables which had adequate cell count. A *p*-value < 0.05 and clinical importance of variables were used to select candidate variables for multivariable logistic regression to avoid over-fitting of the model. Multicollinearity among independent variables was checked in a linear regression model. After multivariable logistic regression, a *p*-value < 0.05 was used to declare statistical significance. Adjusted Odds Ratio (AOR) and 95% CI were used to report the strength of association between outcome (any D&A) and independent variables.

### Data quality control

Questionnaire translated to local language was back-translated to ensure consistency of translation. Data collectors were trained and data collection tool was pre-tested on 5% of a sample before actual data collection. The supervisor checked filled questionnaires for accuracy and completeness on a daily basis. The supervisor replaced grossly incomplete filled questionnaires if the participants had left the hospital or returned to data collectors to fill the incomplete sections if the participants not yet left the hospital.

### Dissemination plan

The finding of this study was submitted to Research and Graduate Studies Coordinating Office, Jimma University.

### Operational definition

● Skilled providers or skilled health personnel or skilled birth attendants are health professionals who are educated and trained to national or international standards [2]. They are qualified to:

(i) Give evidence-based, human-rights-based, quality, socio-culturally acceptable and self-respectful care to women and newborns;
(ii) Help with physiological processes during labor and delivery to ensure a clean and positive childbirth experience;
(iii) Diagnose and treat or refer women and/or newborns with complications.

● For a specific category of abuse and disrespect with more than one verification criterion, a woman was labeled “abused and disrespected” in that category if she was abused and disrespected in at least one of the verification criteria during childbirth.

● If a mother was identified as disrespected and abused in at least one of the seven categories, she was considered “disrespected and abused”.

## Results

### Baseline characteristics

Regarding socio-demographic characteristics, the majorities (220, [75.8%]) were in the age group 20–29 years, 217 (74.8%) were Muslim and 271 (93.4%) were married. Nearly half (142, [49%]) had a primary level of education. The majorities (232, [80%]) were housewives and 175 (60.3%) were from outside Jimma town. Nearly half (128, [44.1%]) of the respondents were in the first two (lowest and second) households’ monthly income quintiles. Concerning obstetric characteristics, more than half (150, [51.7%]) were para one and almost all (285, [98.3%]) had ANC for current pregnancy. The majorities (166, [57.2%]) had no experience of institutional delivery. Regarding services, most (250, [86.2%]) stayed in the hospital at most 24 h. More than half (158, [54.5%]) were attended by at most 2 professionals and most (237, [81.7%]) were attended by male providers (Table 1).

**Table 1:** Baseline characteristics of respondents, JUMC, Oct to Dec 2016

Baseline characteristics		Number (%)	Baseline characteristics		Number (%)
Age (years)	15–19	24 (8.3)	Parity	1	150 (51.7)
	20–24	112 (38.6)		2–3	107 (36.9)
	25–29	108 (37.2)		> = 4	33 (11.4)
	> = 30	46 (15.9)	History of ANC use during current pregnancy	yes	285 (98.3)
Religion	Muslim	217 (74.8)		no	5 (1.7)
	Orthodox	55 (19.0)	History of previous institutional birth	no	166 (57.2)
	Protestant	17 (5.9)		yes	124 (42.8)
	Others	1 (0.3)	Length of hospital stay	≤ 24 h	250 (86.2)
Marital status	Married	271 (93.4)		> 24 h	40 (13.8)
	Others	19 (6.6)	Number of health professionals attended the mother	1–2	158 (54.5)
Educational status	No formal education	67 (23.1)		> 2	132 (45.5)
	Primary school	142 (49.0)	Sex of main health provider who attended a mother	Male	237 (81.7)
	Secondary and above	81 (27.9)		Female	53 (18.3)
Occupation	Housewife	232 (80.0)	Faced birth complication/s during current labor	no	232 (80.0)
	Employee	39 (13.4)		yes	58 (20.0)
	Merchant	6 (2.1)	Income quintiles	Lowest	81 (27.9)
	Student	5 (1.7)		Second	47 (16.2)
	Others	8 (2.8)		Middle	73 (25.2)
Residential address	Outside Jimma	175 (60.3)		Fourth	39 (13.4)
	Jimma town	115 (39.7)		Highest	50 (17.2)

### Prevalence of disrespect and abuse (D&A)

**Fig. 1:**
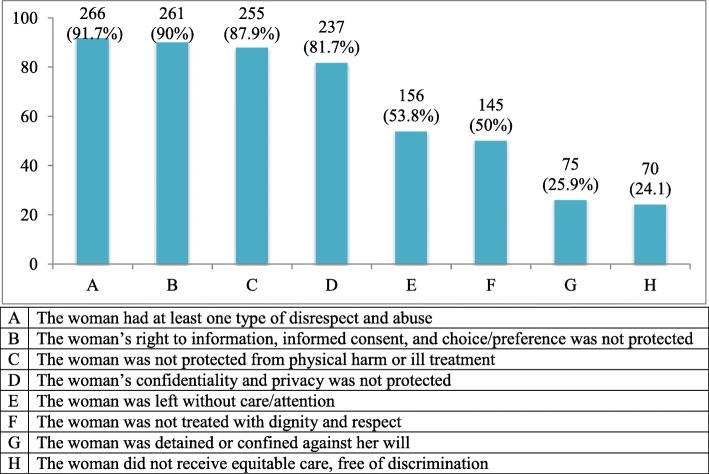
Overall and category specific prevalence of disrespect and abuse, JUMC, Oct to Dec 2016

The overall prevalence (at least one form of disrespect and abuse) was 91.7% (266/290; 95%CI: 0.879, 0.946). The woman’s right to information, informed consent, and choice/preference were not protected in 261 (90%) of mothers. More than four-fifths (255, [87.9%]) of women were not protected from physical harm or ill-treatment during labor and delivery. Similarly, the woman’s confidentiality and privacy were not protected in more than four-fifths of mothers (Fig. 1).

Most women (218, [75.2%]) were not given the care in a culturally appropriate way by the care providers. Similarly**,** the care providers did not encourage the client to ask questions in most cases (220, [75.9%]) and most mothers (232, [80.0%]) reported that care provider didn’t introduce him/herself during childbirth. The provider also did not explain to the client what was being done and what to expect throughout labor and birth in more than half of the cases (150, [51.7%]) and did not give her periodic updates on status and progress of labor in nearly half of the cases (143, [49.3%]). In nearly two-thirds of clients (185, [63.8%]), the provider did not obtain consent or permission prior to any procedure. Again, the provider did not use curtains or other visual barriers to protect the client in most cases (237, [81.7%]) (Table 2).

**Table 2:** Distribution of types of disrespect and abuse reported by mothers during child birth, JUMC, Oct to Dec 2016

Categories of disrespect and abuse	Types of disrespect and abuse (*n* = 290)	Number (%)
Physical harm or ill-treatment	mother did not cared for in a culturally appropriate way	218 (75.2)
	mother denied food/fluid without medical indication	108 (37.2)
	mother did not receive pain-relief as necessary	103 (35.5)
	mother and newborn were separated without medical indication	35 (12.1)
	mother was physically confined	7 (2.4)
	physical force was used (e.g. slapping/hitting the mother)	7 (2.4)
Non-consented care	service provider did not introduce him/herself to the mother	232 (80.0)
	mother was not encouraged to ask questions	220 (75.9)
	consent or permission prior to any procedure not obtained	185 (63.8)
	service provider did not explain what is being done and expected outcome during labor and birth	150 (51.7)
	periodic updates on status and progress of labor not given	143 (49.3)
	service provider did not answer questions promptly, politely and truthfully	129 (44.5)
	mother not allowed to move about during labor	111 (38.3)
	mother not allowed to take position of choice during childbirth	32 (11.0)
Non-confidential care	curtains or other visual barriers not used	237 (81.7)
Non-dignified care	service provider did not speak politely	95 (32.8)
	mother was insulted, intimidated, threaten, or coerced	55 (19.0)
Discrimination on specific grounds/ characteristics	mother shown disrespect based on religion or ethnicity or place of residence, etc.	47 (16.2)
	a language or language-level that mother cannot understand was used	39 (13.4)
Abandonment or denial of care	provider did not arrive quickly when called	105 (36.2)
	mother was not encouraged to call provider if needed	85 (29.3)
	mother was left alone or unattended	56 (19.3)
Detention or confinement in facilities	mother was delayed in health facility against her will	75 (25.9)
Overall prevalence of disrespect and abuse	mothers who faced disrespect and abuse in at least one of the seven categories	266 (91.7)

### Factors associated with disrespect and abuse

The association of maternal socio-demographic characteristics, reproductive factors and service-related factors with experience of disrespect and abuse during facility-based delivery was examined. The strength of the relationship was quantified using Odds Ratio (OR) and 95% confidence interval. Accordingly, marital status, educational level, parity and sex of health provider had a statistically significant association with disrespect and abuse during facility-based childbirth. A respondent who was non-married (either single or widowed or divorced) was more than 95% lower likely [AOR: 0.046; 95%CI: (0.009,0.222) to report disrespect and abuse than married. Clients with secondary education had more than three times higher [AOR: 3.25; 95% CI: (1.028,10.272)] chance to report disrespect and abuse compared to the respondent with primary or no formal education. A para two or above woman was nearly 72% lower likely [AOR: 0.283; 95%CI: (0.067,0.762)] to report disrespect and abuse compared to para one woman. A mother whose delivery was attended by female care provider was more than 92% lower likely [AOR: 0.076; 95%CI: (0.026,0.224)] to report disrespect and abuse compared to a mother whose delivery was attended by a male provider (Table 3).

**Table 3:** Association between client baseline characteristics and reported disrespect and abuse (D&A) during child birth, JUMC, Oct to Dec 2016

		Any D&A		COR (95% CI)	AOR (95% CI)
		Yes	No		
Age	< 25	124	12	0.230 (0.029, 1.817)	
	25–29	97	11	0.196 (0.025,1.565)	
	≥ 30	45	1	1	
Religion	Muslim	201	8	1	
	Non Muslim	65	16	0.162 (0.066,0.395)	
Marital status	Married	253	18	1	1
	Others	13	6	0.154 (0.052,0.453)	0.046 (0.009,0.222)*
Education	No formal education/ primary school	144	19	1	1
	Secondary and above	122	5	3.219 (1.168,8.877)	3.25 (1.028,10.272)*
Occupation	House wife	215	17	1	
	Others	51	7	0.576 (0.213,1.736)	
Household monthly income (quintile)	Lowest +2nd			1	
	Middle			1.279 (0.426,3.836)	
	4th + Highest			0.952 (0.367,2.471)	
Residence	Jimma town	102	13	0.526 (0.227,1.219)	
	Outside Jimma	164	12	1	
Parity (including current one)	1	146	8	1	1
	> 1	120	16	0.411 (0.170, 0.993)	0.283 (0.093,0.862)*
ANC utilization	Yes	262	23	1	
	No	4	1	0.351 (0.033,18.034)	
Length of stay in the hospital	≤ 24 h	228	22	1	
	> 24 h	38	2	1.833 (0.421,16.692)	
Index birth complication	Yes	56	2	2.933 (0.685,26.411)	
	No	210	22	1	
Number of health professionals who attended the mother	1–2	139	19	1	
	> 2	127	5	3.472 (1.259, 9.572)	3.011 (0.838,10.823)
Sex of main health provider who attended mother	Male	228	9	1	1
	Female	38	15	0.10 (0.041,0.245)	0.076 (0.026,0.224)*
Someone other than concerned health provider had got access to see you during labor	Yes	61	3	2.083 (0.601,7.220)	
	No	205	21	1	

## Discussion

This study investigated the prevalence of disrespect and abuse faced by women during facility-based childbirth at Jimma University Medical Centre. Both verbal report (anecdote) and literature indicated that clients are often discriminated on the grounds of race, ethnicity, religion, age, socio-economic, and HIV status [17, 18]. This study also assessed the relationship between D&A and socio-demographic/economic characteristic (age, religion, marital status, educational level, occupation, household monthly income, residence), obstetric characteristics (parity, AN utilization current birth complication), length of stay in the hospital, number of health professionals who attended the mother, sex of main provider and whether someone other than concerned health provider had got access to see the mother during labor.

The overall prevalence of disrespect and abuse (91.7%) observed in this study was higher than 81.8% prevalence reported by the study conducted in Addis Ababa, Ethiopia [21]. This could be because of the difference in settings where the culture of participants and composition of professionals caring for mothers differ. Though final year undergraduate medicine students also involved in facility-based childbirth attendance in both setups, the difference in how they were involved might have increased the magnitude of the problem in our setup. Three-fourths of respondents in our study were Muslims who don’t want to be attended by male providers because of religious interest and culture of the surrounding community. Contrary to this, more than four-fifths of deliveries were attended by male providers. This contradiction might also have increased the report of disrespect and abuse in our setup.

Our finding was also more than four times higher than 19.5, 20 and 21% prevalence reported by studies done in Tanzania [20], Kenya [19] and Ethiopia [22] respectively. It was also more than six times higher than 15% prevalence reported by another study done in Tanzania [24]. Unlike our setup, participants might have normalized or under-reported disrespect and abuse during an immediate postpartum interview (courtesy bias) in both setups as evidenced by the increase of the prevalence to 70% during community follow up interview of Tanzanian study [24]. Kenyan and Tanzanian studies also included a significant proportion of mothers who gave birth by Cesarean section [19, 20]. This might have led to the underestimation of prevalence as mothers are consented and better cared for (e.g. received anesthesia for pain) in such situation and thus, odds was reduced [19]. Providers have greater control over timing and setting of Caesarean section births and may perceive these cases as more serious, therefore behaving more professionally with the patient. A study conducted in Ethiopia was a health center based where client load was much lower and students were not involved in service delivery. Our finding was; however, lower than 98% prevalence reported by the study undertaken in Nigeria [23].

Three-fourths of mothers were not given the care in a culturally appropriate way by the care provider which was more than eight times higher than the report of study from Addis Ababa, Ethiopia [21]. In more than three-fourths of the cases, the care provider did not encourage the client to ask questions and didn’t introduce him/herself during childbirth similar to the report of studies conducted in Ethiopia [21, 22]. The provider did not explain to the client what to be done and what to expect throughout labor and birth and did not give periodic updates on the status and progress of labor in half of the cases. This is higher than the finding of the study undertaken in Addis Ababa, Ethiopia [21]. In two-thirds of clients, the provider did not obtain consent or permission prior to any procedure similar to the report of a health center based study conducted in Ethiopia [22] and Nigeria [23] but, higher than the report of a study done in Addis Ababa, Ethiopia [21] and Tanzania [20, 24]. The provider did not use curtains or other visual barriers to protect the client in four-fifths of the cases which was higher than the report of studies from Ethiopia [21, 22] and Tanzania [24].

In general, this figure indicates a worrisome picture of the quality of care during labor and delivery in the study facility. This is because, in many urban areas of resource-limited countries, improved access to services (especially in hospitals) has led to an increase in women seeking facility-based care during childbirth. As a result, many urban hospitals including our study facility had high patient flow and faced significant resource and staff shortages which are likely to be one of the key drivers of disrespect and abuse.

Women who attended secondary education or greater were more likely to report D&A similar to the finding of Tanzanian study [20]. This is likely due to a combination of higher expectations of care quality and greater empowerment to report abuse. Those with experienced births (para two or above) were less likely to report D&A similar to the finding of Tanzanian study [20, 22]. This may indicate quicker and easier deliveries and/or greater resistance to or normalization of abusive remarks or behavior. It may also reflect anxiety about the birth experience as well as a greater need for care of those with first birth.

Previous studies have reported that married women were less likely to have experienced disrespect than unmarried women may be because of negative attitude among medical staff towards unmarried pregnant women [20]. However, we found that married women were more likely to have experienced disrespect than unmarried women may be because of more expectation of respectful care by this group.

Unlike previous studies, stay in the facility for delivery [20] and childbirth complications [19, 22] were not found associated with reporting D&A. However, a woman whose delivery was attended by female healthcare provider was less likely to report D&A. This could because of religious and cultural preference. In the current study, three-fourths of participants were Muslims and most were housewives which could be the reason for preferring female professionals.

## Limitations

This study had some limitations. Disrespect and abuse is a complex concept to measure. Our assessment was based on self-report rather than objectively measuring the frequency of disrespectful and abusive care in the facility. However, given that disrespect and abuse are determined by women’s own view of what is disrespectful and abusive, we believe that a self-reported measure is appropriate. Some studies have complemented exit interviews with community interviews conducted four to 10 weeks post-delivery to compare reported D&A [20, 24]; others complemented it with observation [24]. Unfortunately, the study budget did not allow us for this method approach. Our analysis also did not include details of provider and facility-level factors as contributors to disrespect and abuse and thus, future research should explore these. Although interviews were conducted at the time of hospital discharge, the fact that women were asked about their care in the place where they give birth and by staff employed by the hospital, albeit not part of their care provision, may still induce social desirability bias.

## Conclusion

The finding that nine out of 10 women experienced abuse or disrespect during facility-based delivery shows a health system in crisis. Abuse during childbirth may have far-reaching consequences in future health care utilization. A key implication of this finding is that efforts to increase facility-based delivery must address disrespect and abuse to ensure higher utilization by women and to safeguard women’s fundamental rights during facility delivery. Making facility-based deliveries attended by female providers may reduce the problem.

## Abbreviations

ANC: Antenatal Care
MDG: Millennium Development Goal
MMR: Maternal mortality ratio
EDHS: Ethiopian Demographic and Health Surveillance
D&A: Disrespect and abuse
JUMC: Jimma University Medical Centre
